# PET/MRI of glucose metabolic rate, lipid content and perfusion in human brown adipose tissue

**DOI:** 10.1038/s41598-021-87768-w

**Published:** 2021-07-22

**Authors:** Elin Lundström, Jonathan Andersson, Mathias Engström, Mark Lubberink, Robin Strand, Håkan Ahlström, Joel Kullberg

**Affiliations:** 1 Department of Surgical Sciences, Section of Radiology, Uppsala University, Uppsala, Sweden; 2 GE Healthcare, Waukesha, WI USA; 3 Medical Physics, Uppsala University Hospital, Uppsala, Sweden; 4 Department of Information Technology, Uppsala University, Uppsala, Sweden; 5 Antaros Medical, BioVenture Hub, Mölndal, Sweden

**Keywords:** Magnetic resonance imaging, Positron-emission tomography, Fat metabolism

## Abstract

This study evaluated the MRI-derived fat fraction (FF), from a Cooling-reheating protocol, for estimating the cold-induced brown adipose tissue (BAT) metabolic rate of glucose (MR_glu_) and changes in lipid content, perfusion and arterial blood volume (V_A_) within cervical-supraclavicular fat (sBAT). Twelve volunteers underwent PET/MRI at baseline, during cold exposure and reheating. For each temperature condition, perfusion and V_A_ were quantified with dynamic [^15^O]water-PET, and FF, with water-fat MRI. MR_glu_ was assessed with dynamic [^18^F]fluorodeoxyglucose-PET during cold exposure. sBAT was defined using anatomical criteria, and its subregion sBAT_HI_, by MR_glu_ > 11 μmol/100 cm^3^/min. For all temperature conditions, sBAT-FF correlated negatively with sBAT-MR_glu_ (ρ ≤ − 0.87). After 3 h of cold, sBAT-FF decreased (− 2.13 percentage points) but tended to normalize during reheating although sBAT_HI_-FF remained low. sBAT-perfusion and sBAT-V_A_ increased during cold exposure (perfusion: + 5.2 ml/100 cm^3^/min, V_A_: + 4.0 ml/100 cm^3^). sBAT-perfusion remained elevated and sBAT-V_A_ normalized during reheating. Regardless of temperature condition during the Cooling-reheating protocol, sBAT-FF could predict the cold-induced sBAT-MR_glu_. The FF decreases observed after reheating were mainly due to lipid consumption, but could potentially be underestimated due to intracellular lipid replenishment. The influence of perfusion and V_A_, on the changes in FF observed during cold exposure, could not be ruled out.

## Introduction

The main function of white adipose tissue (WAT) is to store energy in the form of intracellular lipids (fat). Brown adipose tissue (BAT), however, uses substrates such as lipids and glucose to generate heat in a process called non-shivering thermogenesis (NST), upon metabolic activation e.g. by cold exposure^1,2^. Negative associations between adiposity and cold-induced [^18^F]fluorodeoxyglucose ([^18^F]FDG) uptake, assessed with positron emission tomography (PET)^3,4^, and positive effects on glucose metabolism in subjects exhibiting visible BAT in PET examinations during 5–8 h cold exposure^5^, indicate BAT as a possible target for treatment of obesity and diabetes. This therapeutic potential has made BAT focus of a large research interest but to determine its capacity, safe methods providing reliable estimations of BAT amount and metabolic activity are needed. Currently, there is no non-invasive approach for estimating BAT amount in terms of brown adipocyte quantity^6^. In-vivo, BAT metabolic activity is typically studied with respect to glucose metabolism using [^18^F]FDG-PET combined with computed tomography ([^18^F]FDG-PET/CT)^6^, which yields either the physiologically semi-quantitative standard uptake value (SUV) from conventional static acquisition or quantitative estimates, e.g. the metabolic rate of glucose (MR_glu_), from dynamic acquisition. Quantitative estimates are generally preferable when comparing glucose metabolism between subjects with different body composition^7^ as SUV overestimates the assessment in overweight/obese subjects. Imaging is conducted during warm and/or cold conditions, with the latter promoting increased [^18^F]FDG uptake, assumed to be accompanied by an overall increase in BAT metabolic activity and NST. Previous studies have reported higher CT Hounsfield units (HUs) of adipose tissue regions with high [^18^F]FDG uptake compared to low [^18^F]FDG uptake^8,9^. In addition, the CT HUs of supraclavicular BAT have been observed to increase during 3 h of cold exposure, interpreted as a result of lipid consumption in BAT during NST^10^. Water-fat MRI, introduced as a non-ionizing imaging alternative for BAT, has been used to quantify a corresponding cold-induced decrease in fat fraction (FF) within the cervical-supraclavicular fat depot^11–13^, with FF calculated as the MR signal from fat divided by the total signal from water and fat. In these studies, PET reference measurements of BAT were lacking, and as a result, the association between e.g. [^18^F]FDG uptake and changes in FF could not be determined. Another study, performed with PET/CT and MRI of supraclavicular adipose tissue, failed to show a significant relationship between cold-induced [^18^F]FDG-SUV and changes in FF between baseline and cold exposure^14^. Whether a more quantitative PET approach would discern such an association has not yet been studied. Dynamic [^15^O]water-PET studies have reported increased BAT perfusion during cold exposure^15–17^ and a cold-induced increase in BAT water content due to increased tissue arterial blood volume (V_A_)^17^ that incorrectly might be interpreted as a decrease in absolute fat content in the FF measurements. We have previously proposed a Cooling-reheating protocol, involving MRI at baseline, during cold exposure and during reheating, for isolating the contribution of lipid consumption (i.e. decrease in absolute fat content) to the cold-induced decrease in FF^11^. The protocol concept was based on assumptions that cold-induced increases in perfusion (and blood volume) would normalize relatively fast during subsequent reheating whereas cold-induced decreases in lipid content would remain low^11^. When applying the Cooling-reheating protocol, a sustained low FF at the end of 30 min of reheating was observed, indicating lipid consumption, rather than altered perfusion, as the primary cause of the preceding cold-induced decrease in FF^11^. However, verification of perfusion as a fast-regulated process, e.g. with reference perfusion measurements from PET, has not yet been performed. In a previous PET/CT study, peak radioactivity from [^11^C]acetate in BAT was used as index of perfusion and BAT CT HUs were used to indicate lipid content in subjects exposed to 3 h of cold exposure during two separate visits. One of the visits involved ingestion of nicotinic acid for inhibition of intracellular triglyceride lipolysis and the other visit served as control^18^. The results indicated cold-induced increases in BAT CT HUs to mainly be due to lipid consumption and not to increased perfusion, which is in line with our previous study^11^. However, quantification of the effects of cold exposure on BAT perfusion and lipid content, using gold-standard procedures ([^15^O]water-PET and water-fat MRI, respectively), would strengthen previous studies and deepen our knowledge of BAT physiology.

Unlike PET and CT, MRI does not expose the subjects to ionizing radiation and could therefore be of particular use in BAT studies including repeated imaging and/or imaging of children. Water-fat MRI has been used for characterizing BAT and distinguishing it from WAT (typically represented by subcutaneous adipose tissue, SAT), based on its presumed higher intracellular water content and richer vascularization^19,20^. Numerous studies have investigated the association between FF and BAT glucose metabolism (assessed from either static or dynamic [^18^F]FDG-PET) and/or the difference in FF between subjects/scans/regions exhibiting high and low BAT-related [^18^F]FDG uptake^14,20–27^. Supraclavicular-FF, estimated with MRI or magnetic resonance spectroscopy (MRS) during warm^23,24^ and cold^23,26^ conditions, has shown a negative correlation with cold-induced [^18^F]FDG-SUV^26^ and MR_glu_^23,24^, indicating FF as suitable for BAT assessment regardless of the tissue glucose metabolic status. Imaging during warm and cold conditions were performed at separate visits^23^ and changes in FF per se could therefore not be investigated. Studies targeting the differences in FF between cervical-supraclavicular regions of high and low BAT glucose metabolism are few, whereof one has reported lower FF in regions of higher [^18^F]FDG-SUV^27^, and another, no general difference except for in subjects with low body mass index (BMI)^25^. Such regional differences have not yet been targeted with a more quantitative (dynamic) PET approach.

This study aimed to evaluate: 1) The degree to which FF in cervical-supraclavicular fat (i.e. suspected BAT, hereafter denoted sBAT), measured during a Cooling-reheating protocol, is associated with the cold-induced BAT metabolic rate of glucose (MR_glu_), assessed with dynamic [^18^F]FDG-PET. 2) Whether FF measurements from the Cooling-reheating protocol can be used for detecting changes in lipid content and perfusion (including arterial blood volume, V_A_) related to cold-induced MR_glu_ in BAT, using [^15^O]water-PET to obtain reference measurements of perfusion and V_A_.

## Materials and methods

### Subjects

After study approval by the Regional Ethical Review Board in Uppsala and the Radiation Protection Committee in Uppsala, twelve adult volunteers were recruited, mainly through advertisement. Informed written consent was obtained at the beginning of the study and all methods were carried out in accordance with the relevant guidelines and regulations. Subject characteristics: 7 males, age 27.4 ± 6.1 (21–42) years, weight 72.3 ± 16.1 (54.6–108.0) kg, BMI 23.5 ± 4.1 (18.0–34.2) kg/m^2^, waist circumference 81.6 ± 12.4 (61.5–102.0) cm; mean ± standard deviation (range). Participants were screened for exclusion criteria according to brown adipose reporting criteria in imaging studies (BARCIST) 1.0^28^ (i.e. the use of β-blockers and β-adrenergic agonists, weight change of  > 5% within 3 months, habitual tobacco use, habitual excessive alcohol use, pregnancy and plasma glucose > 11 mM). In addition, subjects otherwise unsuited for PET/MRI and/or with self-reported tendency of impaired blood circulation in extremities during cold conditions were excluded. In line with BARCIST 1.0^28^, subjects were instructed to refrain from high-fat food and caffeine 24 h prior to the study visits, to fast over night from 22:00 the preceding evening and to avoid intense physical activity 48 h before each visit. They were also instructed to refrain from donating blood within one month of the study, to avoid cold exposure before the investigations (e.g. during the journey to the hospital) and to drink 0.5 l of water in the morning of the investigations (to decrease the biological half-life of [^18^F]FDG). For subjects with a suspected on-going infection, the investigations were postponed.

### Preparation and basic body measurements

Initial preparations included information to the subject, verification of subject preparations, ruling out of exclusion criteria, obtaining of informed written consent, interviewing of subject, dressing in standardized clothing and conduction of basic body measurements (weight, height and waist circumference). A heat fan was used to maintain a comfortable ambient temperature during these preparations. Throughout the study visits, the subjects were dressed in standardized clothing consisting of underwear, socks and patient gown. Subject weight and height were obtained with scales and stadiometers, respectively. Waist circumference was measured midway between the lowest rib and the superior border of the iliac crest on a standing subject. The body mass index (BMI) was calculated according to $\text{BMI = weight/}{\text{height}}^{2}$.

### Protocol

**Figure 1:**
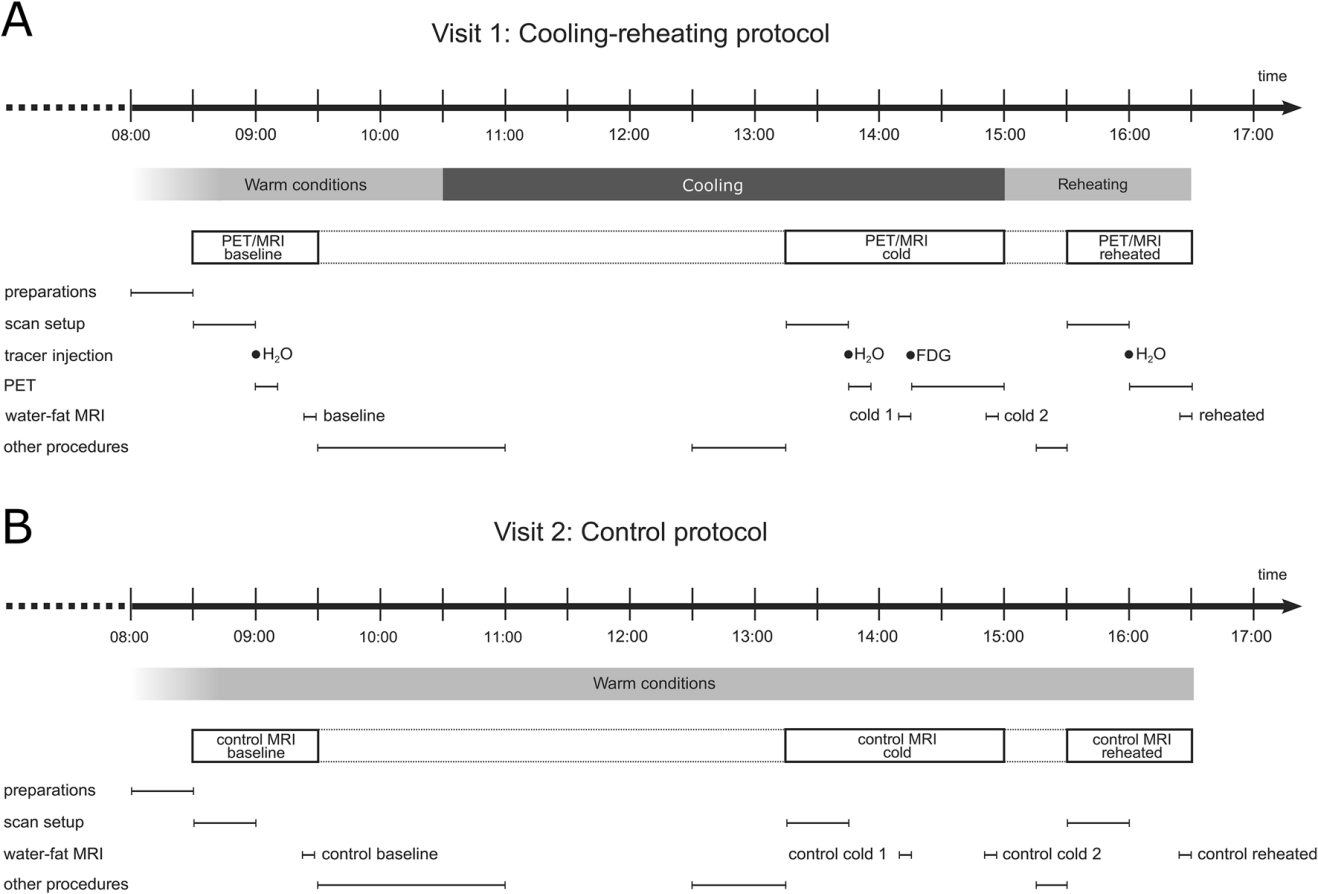
Schematic overview of the study protocols. **(A)** Cooling-reheating protocol and **(B)** Control protocol. Timings of procedures are indicated. *Other procedures* denote measures not included in the present work. H_2_O denotes [^15^O]water and FDG denotes [^18^F]FDG.

The study comprised two visits on separate days, typically within 2–4 weeks of each other (Fig. 1). Visit 1 involved three PET/MRI examinations during a cooling-reheating intervention. MRI at baseline and during cold exposure were used for e.g. assessing the cold-induced change in sBAT-FF, potentially related to MR_glu_ in BAT. MRI during subsequent reheating aided in separating between the contributions from perfusion (including V_A_) and lipid content to the preceding cold-induced change in sBAT-FF. The distinction between the effects of perfusion and lipid content was based on the assumptions that perfusion and V_A_ would normalize relatively fast during reheating (~ 1 h in this study) whereas a decreased lipid content would persist during the same reheating time. Dynamic [^18^F]FDG-PET during cold exposure provided a measurement of cold-induced MR_glu_ in BAT. It should be emphasized that sBAT-MR_glu_ only targets the glucose metabolism of the tissue, which is not equivalent to its thermogenic state. In this study, however, sBAT-MR_glu_ was assumed to be positively related to BAT NST, as supported by previous studies^24,29^. [^15^O]water-PET at baseline, during cold exposure and reheating yielded references of temperature-induced changes in sBAT-perfusion and sBAT-V_A_. PET and MRI were performed with a clinical whole-body 3.0 T PET/MR system (Signa PET/MR, GE Healthcare, Waukesha, WI, USA). Visit 2 served as control to visit 1 and was performed only under warm conditions and without PET (to restrict the ionizing radiation exposure).

#### Visit 1

This visit started with initial preparations (see “Preparation and basic body measurements” section). Baseline PET/MRI was performed during warm conditions with the subject wearing a vest (CoolFlow Heavy Duty cooling vest, Polar Products Inc, Stow, OH, USA) containing tubing filled with non-circulating water of room temperature. Due to the challenging logistics associated with short-lived ^15^O, scan setup was initiated ~ 30 min prior to imaging. The baseline scan consisted of a dynamic [^15^O]water-PET perfusion measurement and a simultaneous MRI protocol including a water-fat MRI sequence. After the baseline scan, some study-related procedures outside the scope of this work (labelled *other procedures* and partly presented previously^30^) were performed. Subsequently, the subject was exposed to cold while seated inside an air-conditioned room (~ 16 °C), wearing the same vest but this time perfused with cold water (initially 4 °C but increased when the subject reported tendency to shiver). After ~ 1 h 30 min of rest and 45 min of other procedures, scan setup for the second PET/MRI was initiated. It was conducted during maintained cooling, using the water-perfused vest, and started with a dynamic PET perfusion measurement and a simultaneous MRI protocol (identical to those performed at baseline), and ended with a dynamic [^18^F]FDG-PET measurement and a single water-fat MRI sequence. The first water-fat MRI during cold exposure is hereafter denoted *cold scan 1* and the second *cold scan 2*. After PET/MRI during cold exposure, the subject was reheated while seated during ~ 30 min in a dedicated room, using a heating fan, a ceiling-mounted heater, the water-perfused vest (warm water) and four heating pads. The last ~ 15 min of reheating were devoted to other procedures and subsequently, the subject underwent the last PET/MRI during maintained reheating (vest, four heating pads placed at groin). The reheated scan consisted of a PET perfusion measurement with a prolonged acquisition (see “Image acquisition” section). The additional data acquired were used during post-processing for removal of residual [^18^F]FDG in the perfusion compartment modelling. The MRI protocol was identical to that performed at baseline. The water-fat MRI data of visit 1 of the first subject were acquired with a pilot sequence (see “Image acquisition” section).

#### Visit 2

This visit was very similar to visit 1 (Fig. 1), except for the absence of radiotracer administration, PET and cooling-reheating intervention. Care was taken to match the MRI acquisition time points of visit 2 to those from visit 1.

### Image acquisition

For each PET/MRI, care was taken to position the subject straight with symmetrical shoulders, for more consistent image coverage in all examinations.

#### PET

The venous whole-blood glucose level (${\text{C}}_{\text{glu}}^{\text{WB}}$) was measured using Contour XT (Bayer Healthcare, Basel, Switzerland) before and after PET/MRI during cold exposure. A typical mean plasma-to-whole-blood ratio of 1.1 was used to convert ${\text{C}}_{{{\text{glu}}}}^{{{\text{WB}}}}$ to plasma glucose level (${\text{C}}_{\text{glu}}^{\text{P}}$)^31^. In addition, venous blood was sampled prior to the PET/MRI during cold exposure and analysed with respect to plasma glucose level (${\text{C}}_{\text{glu}}^{\text{P2}}$) using standard operating procedures at the central laboratory of the hospital. The PET field of view (FOV) was along the superior-inferior direction centred on the supraclavicular fat depot. The perfusion measurements included an intravenous injection of 600 MBq of [^15^O]water at the start of a 10 min dynamic scan with time frames 1 × 10, 8 × 5, 4 × 10, 2 × 15, 3 × 20, 2 × 30 and 6 × 60 s. During reheating, an additional 60 s frame was acquired immediately prior to injection and another 60 s frame was acquired 20 min after injection. These two 60 s frames were used for subtracting the residual [^18^F]FDG radioactivity from the dynamic [^15^O]water images. Three approaches to [^18^F]FDG background subtraction were investigated, assuming either constant [^18^F]FDG, a linearly changing [^18^F]FDG, or an exponentially changing [^18^F]FDG contribution during the [^15^O]water scan.

The MR_glu_ measurement included the intravenous injection of 3 MBq/kg [^18^F]FDG simultaneously with the start of a 45 min dynamic scan with time frames 1 × 10, 8 × 5, 4 × 10, 2 × 15, 3 × 20, 2 × 30, 6 × 60, 4 × 150 and 5 × 300 s. All radioactivity injections were performed with a contrast medium injector as a fast bolus (10 ml at 1 ml/s followed by 30 ml saline at 2 ml/s). Images were reconstructed into a 128 × 128 × 89 matrix with 500 mm transaxial FOV using time-of-flight ordered subset expectation maximization (3 iterations, 28 subsets) including point spread function recovery, all appropriate corrections for randoms, scatter etc. and a 5 mm Gaussian post-filter. Attenuation correction was based on a built-in dual-echo water-fat MRI sequence.

#### MRI

The MRI protocol consisted of a series of sequences acquired using a 19-element head-neck unit (HNU) receive coil (GE Healthcare). The sequence used in this work was a 4 min 52 s multi-echo 3D gradient echo sequence acquired in free breathing. To reduce respiratory artefacts, the subjects were instructed to breathe shallowly. Scan parameters: axial acquisition, repetition time/echo time 1/echo time spacing = 13.8/1.70/0.65 ms, 15 unipolar echoes acquired as five consecutive time-shifted readouts of echo train length 3, flip angle = 4°, receive bandwidth =  ± 142.86 kHz, parallel imaging acceleration = 1.5 (ARC) in anterior–posterior and superior-inferior direction, FOV (right-left × anterior–posterior × feet-head) = 480 × 202 × 76 mm^3^, acquired/reconstructed voxel size = 1.0 × 1.0 × 2.0 mm^3^/0.94 × 0.94 × 2.0 mm^3^, 38 slices, number of signal acquisitions = 1. The flip angle was chosen small to reduce T_1_-weighting. No MRI contrast agents were administered. The first individual investigated was a pilot subject. After visit 1 of this subject, the water-fat MRI sequence was slightly adjusted. To be able to compare the pilot sequence with the new sequence, this subject was imaged using both sequences during visit 2. The scan parameters of the pilot sequence differed from the new according to: repetition time/echo time 1/echo time spacing = 22.5/2.06/1.07 ms, 15 unipolar echoes acquired as three consecutive time-shifted readouts of echo train length 5, flip angle = 7°, field of view (right-left × anterior–posterior × feet-head) = 480 × 202 × 56 mm^3^, 28 slices.

### Image post-processing

#### PET − Input curves

In both the [^15^O]water and [^18^F]FDG images, a nearly circular region of interest (ROI) of diameter ~ 1 cm was manually outlined using Voiager (GE Healthcare), over the ascending aorta in 5–10 consecutive image slices in the frame in which the first pass of the radioactivity bolus was best visible. These ROIs were combined into a volume of interest (VOI) and projected onto all other frames of the dynamic scans to obtain whole-blood time-activity curves (TACs). For [^18^F]FDG, a plasma input curve was computed by multiplying the whole-blood TAC by a typical mean plasma-to-whole-blood ratio of 1.1.^31^.

#### PET − Perfusion

The dynamic [^15^O]water images were projected onto the sBAT VOIs, obtained from MRI (see Supplemental material), resulting in one TAC for each VOI. Perfusion, distribution volume (V_T_) and V_A_ were estimated using non-linear regression of the operational equation of the standard single-tissue compartment model to the first three minutes of the PET data. In addition to these parameters, the model included a fitted parameter accounting for delay of the input function before arrival in the sBAT VOI. Fits were performed using in-house developed software in MATLAB (MathWorks).

#### PET − Metabolic rate of glucose

Net uptake rate (K_i_) images of [^18^F]FDG were computed using a basis function implementation of the irreversible two-tissue compartment model, employing one irreversible basis function and 50 basis functions with logarithmically spaced clearance rates (between 0.02 and 1.0 min^−1^). Parametric K_i_ images were computed using in-house developed software in MATLAB. Thereafter, MR_glu_ maps were calculated according to

1 $${\text{MR}}_{{{\text{glu}}}} = \overline{{{\text{C}}_{{{\text{glu}}}}^{{\text{P}}} }} \cdot {{{\text{K}}_{{\text{i}}} } \mathord{\left/ {\vphantom {{{\text{K}}_{{\text{i}}} } {{\text{LC}}}}} \right. \kern-\nulldelimiterspace} {{\text{LC}}}},$$

where $\overline{{\text{C}}_{\text{glu}}^{\text{P}}}$ corresponds to mean ${\text{C}}_{\text{glu}}^{\text{P}}$ obtained before and after the PET/MRI during cold exposure. LC corresponds to the lumped constant, relating [^18^F]FDG kinetics to glucose kinetics, here assumed to be 1. The MR_glu_ maps were projected onto the sBAT VOIs obtained from MRI (see Supplemental material) and mean MR_glu_ in each VOI was calculated. Due to motion during image acquisition, the dynamic data of one of the subjects were corrected for motion, using rigid image registration, before the MR_glu_ map was estimated. For eight subjects, the MR_glu_ map was spatially registered to the MRI fat signal image, using rigid image registration, for improved spatial correspondence.

#### Water-fat MRI

**Figure 2:**
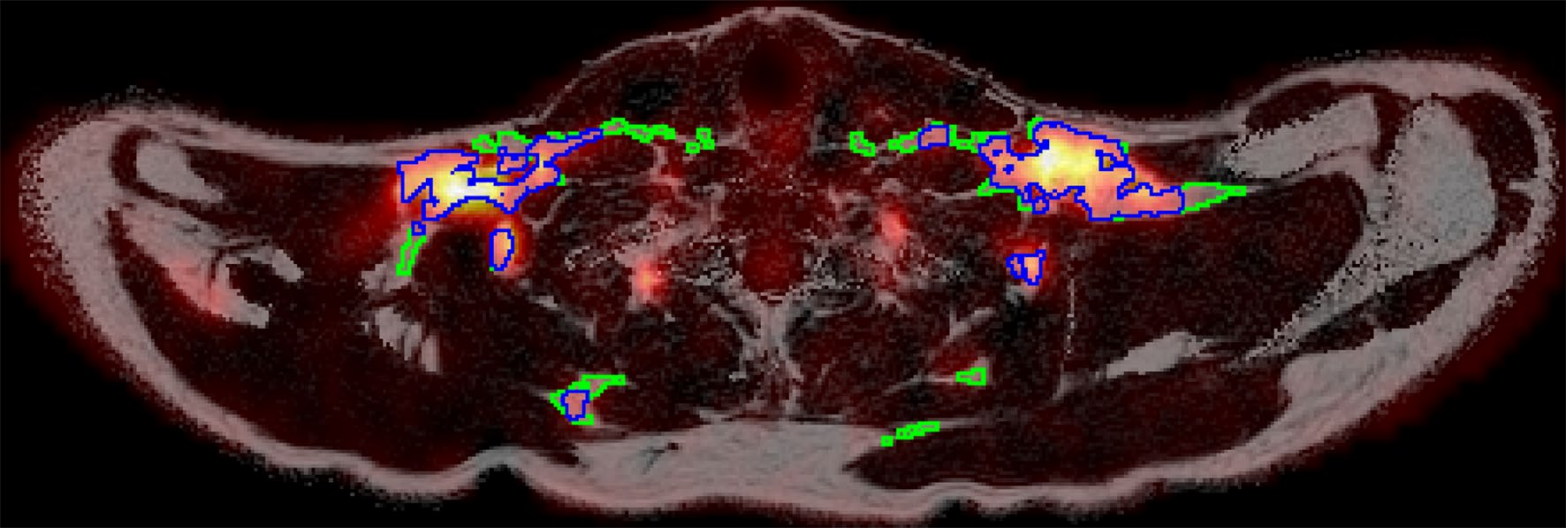
Example of an axial FF map (greyscale) overlaid with the corresponding MR_glu_ map (heat colour scale) and segmentations representing sBAT_HI_ (blue contour) and sBAT_LO_ (green contour).

Reconstruction of water-fat MRI data was carried out using in-house MATLAB software, according to a previously described method^32^. Mean sBAT-FF, sBAT-MR_glu_, sBAT-perfusion and sBAT-V_A_ were obtained from atlas-based segmentation (based on a previously presented method^33^), and mean SAT-FF and SAT-MR_glu_ from automated segmentation with manual corrections (see Supplemental material). Within each sBAT and SAT segmentation, two regions corresponding to high and low MR_glu_ (sBAT_HI_, sBAT_LO_, SAT_HI_ and SAT_LO_) were identified. sBAT_HI_ and SAT_HI_ included voxels with MR_glu_ > 11 μmol/100 cm^3^/min, and sBAT_LO_ and SAT_LO_ included voxels with MR_glu_ ≤ 11 μmol/100 cm^3^/min (see Supplemental material and segmentation examples in Fig. 2).

#### FF, MR_glu_, perfusion and V_A_ estimates

Mean FF and mean MR_glu_ were estimated in sBAT, sBAT_HI_, sBAT_LO_, SAT, SAT_HI_ and SAT_LO_. For comparison, ${\text{C}}_{\text{glu}}^{\text{P2}}$, acquired during cold exposure and measured by the central laboratory of the hospital, was used in Eq. 1 for assessing an alternative MR_glu_ measurement (MR_glu2_) within sBAT and SAT (see Supplemental material). Mean perfusion and V_A_ were only estimated in sBAT. Results related to V_T_ were not included in this work due to the difficulty in reliably estimating V_T_ at low perfusion values, due to the lack of clear patterns in V_T_ with respect to cooling and reheating in initial analyses, and also in order to restrain the content of the manuscript. Mean FF estimates from the pilot water-fat MRI sequence (subject 1 during visit 1) were corrected in order to be comparable to the mean FF of other subjects. The correction was accomplished according to

2 $${\text{mean}}\;{\text{FF}}_{{{\text{corrected}}}} = {\text{mean}}\;\text{FF}_{{{\text{pilot}}}} {-}3.9,$$

where the value 3.9 pp corresponds to the difference in mean sBAT-FF between the two water-fat MRI sequences of visit 2, averaged over the four scans.

### Missing data

Due to protocol development and image quality issues, there were some missing data in this study. Cold scan 2 was not part of the initial protocol and therefore not performed for the first pilot subject. For another subject, the Control protocol was not performed. In addition, the FF map from cold scan 1 of one individual, from cold scan 2 of another individual and from the control baseline scan of a third individual were excluded due to poor image quality. Using an acceptance limit of 100% of the coefficient of variation of the perfusion and V_A_ estimates, perfusion measurements from all three time points (baseline, cold exposure and reheating) were successfully obtained for all twelve subjects whereas the V_A_ measurement from baseline of one subject and reheating of another subject were excluded. The venous blood sampling during cold exposure failed for one of the subjects leading to a missing MR_glu2_ data point (see Supplemental material).

### Statistics and figures

Differences and changes in mean values were evaluated using Wilcoxon signed-rank test. Spearman rank correlation and complementary Pearson correlation (for comparison with literature) were used for associations. *P* < 0.05 was considered statistically significant and *P* < 0.10 as trend. As this was an exploratory study, no correction for multiple comparisons was applied and P-values were interpreted with caution. The coefficient of variation between the baseline FF measurements of the Cooling-reheating protocol and the Control protocol was calculated as an assessment of the precision of the FF estimation. Statistical analysis was performed in RStudio and MATLAB. The figures in this manuscript were prepared using MATLAB, Microsoft Excel for Mac and Inkscape (https://inkscape.org).

## Results

This introductory section provides a summary of the main results. For more details, see following sections and Supplemental material. For each time point during the Cooling-reheating protocol, sBAT-FF was low compared to SAT-FF. Regional differences in sBAT-FF, between sBAT_HI_ and sBAT_LO_, were observed at the end of 3 h of cold exposure but not discernible at baseline and during reheating. Negative correlations between FF and MR_glu_, in sBAT, sBAT_HI_ and sBAT_LO_, were obtained for all temperature conditions. FF of sBAT, sBAT_HI_ and sBAT_LO_ decreased during 3 h of cold exposure but remained low only for sBAT_HI_ during reheating. sBAT-perfusion and sBAT-V_A_ increased during cold exposure. sBAT-perfusion remained elevated whereas sBAT-V_A_ normalized during reheating. Cold-induced changes in sBAT-FF, with respect to baseline, correlated negatively with MR_glu_ and with cold-induced changes in sBAT-V_A_, with respect to baseline.

### Cooling-reheating protocol

#### FF, perfusion and V_A_

FF of SAT, sBAT, sBAT_HI_ and sBAT_LO_ are presented in Table 1A. Perfusion and V_A_ of sBAT are presented in Table 1B,C, respectively. At baseline, sBAT-FF was 4.99 pp lower than SAT-FF (individual sBAT-FF and SAT-FF measurements and their correlation are shown in Fig. S1, Supplemental material) but FF between sBAT_HI_ and sBAT_LO_ did not differ. At cold scan 1, sBAT-FF was 8.21 pp lower compared to SAT-FF and sBAT_HI_-FF was 2.27 pp lower than sBAT_LO_-FF. At cold scan 2, sBAT-FF remained lower than SAT-FF and there was a trend of lower sBAT_HI_-FF compared to sBAT_LO_-FF (*P* = 0.074). During reheating, sBAT_HI_-FF and sBAT_LO_-FF did not differ.

**Table 1:** Measurements from the Cooling-reheating protocol. **(A)** FF within SAT and sBAT, **(B)** perfusion and **(C)** V_A_ in sBAT.

Variable	SAT	sBAT	*(P)* (SAT vs. sBAT)	n	sBAT_HI_	sBAT_LO_	*(P)* (sBAT_HI_ vs. sBAT_LO_)	n
**A**								
Baseline FF	**84.84 ± 5.39**	**79.84 ± 7.07**	**0.003**	12	79.52 ± 7.50	80.54 ± 6.10	0.278	11
Cold 1 FF	**85.96 ± 4.96**	**77.75 ± 8.75**	** < 0.001**	11	**76.91 ± 9.16**	**79.18 ± 7.44**	**0.014**	10
Cold 2 FF	**83.89 ± 5.08**	**76.76 ± 8.53**	**0.004**	10	75.43 ± 8.48	77.72 ± 6.54	0.074	9
Reheated FF	**84.40 ± 5.77**	**78.60 ± 7.70**	**0.009**	12	77.79 ± 7.88	79.33 ± 6.31	0.123	11

Variable	sBAT	n						
**B**								
Baseline perfusion	13.2 ± 9.3	12						
Cold perfusion	18.3 ± 5.9	12						
Reheated perfusion	21.6 ± 12.4	12						

**C**								
Baseline V_A_	2.9 ± 2.0	11						
Cold V_A_	7.3 ± 3.8	12						
Reheated V_A_	2.1 ± 1.5	11						

Mean ± standard deviation. Units: FF, %; perfusion, ml/100 cm^3^/min; V_A_, ml/100 cm^3^. n, number of observations or number of observations included in group comparison where differences in FF between SAT and sBAT and between sBAT_HI_ and sBAT_LO_ were evaluated using Wilcoxon signed-rank test. Statistically significant differences (*P* < 0.05) in bold font.

**Figure 3:**
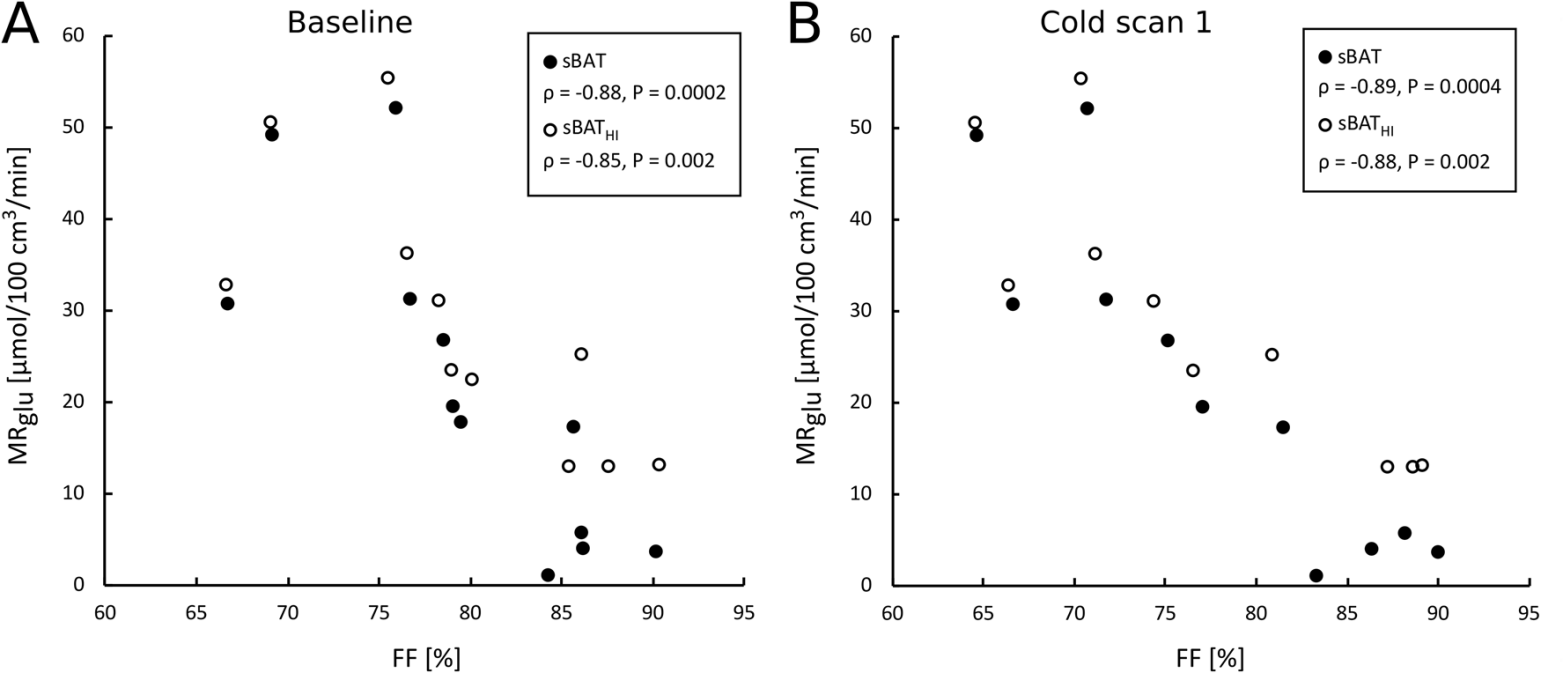
Correlation between MR_glu_ and FF in sBAT and sBAT_HI_ at **(A)** baseline and **(B)** the end of ~ 3 h of cold exposure (cold scan 1). ρ, Spearman rank-order correlation coefficient.

Correlations between MR_glu_ and each of FF and perfusion, during the Cooling-reheating protocol, are provided in Table 2A,B and Fig. 3. For all time points, FF correlated negatively with MR_glu_ within the same region (sBAT: ρ ≤ − 0.87, r ≤ − 0.81; SAT: ρ ≤ − 0.80, r ≤ − 0.69). FF of sBAT_HI_ and sBAT_LO_ correlated with MR_glu_ in a similar fashion. An alternative MR_glu_ assessment (MR_glu2_), based on separate blood samples, provided comparable correlation results with sBAT-FF and SAT-FF (Table S1, Supplemental material). Only sBAT-perfusion estimated during reheating correlated significantly with sBAT-MR_glu_.

**Table 2:** Correlation between MR_glu_ and **(A)** FF and **(B)** perfusion, during the Cooling-reheating protocol.

Variable	*ρ (P)*		r *(P)*		n
**A**					
***Baseline FF***					
sBAT	**− 0.88 (< 0.001)**		**− 0.81 (0.001)**		12
sBAT_HI_	**− 0.85 (0.002)**		**− 0.76 (0.006)**		11
sBAT_LO_	**− 0.81 (0.002)**		**− 0.74 (0.006)**		12
SAT	**− 0.83 (0.002)**		**− 0.71 (0.009)**		12
***Cold 1 FF***					
sBAT	**− 0.89 (< 0.001)**		**− 0.90 (< 0.001)**		11
sBAT_HI_	**− 0.88 (0.002)**		**− 0.87 (< 0.001)**		10
sBAT_LO_	**− 0.85 (0.002)**		**− 0.75 (0.008)**		11
SAT	**− 0.80 (0.005)**		**− 0.69 (0.020)**		11
***Cold 2 FF***					
sBAT	**− 0.90 (< 0.001)**		**− 0.91 (< 0.001)**		10
sBAT_HI_	**− 0.85 (0.006)**		**− 0.85 (0.003)**		9
sBAT_LO_	**− 0.89 (0.001)**		**− 0.79 (0.006)**		10
SAT	**− 0.83 (0.006)**		**− 0.69 (0.029)**		10
***Reheated FF***					
sBAT	**− 0.87 (< 0.001)**		**− 0.87 (< 0.001)**		12
sBAT_HI_	**− 0.83 (0.003)**		**− 0.81 (0.003)**		11
sBAT_LO_	**− 0.82 (0.002)**		**− 0.78 (0.003)**		12
SAT	**− 0.83 (0.001)**		**− 0.74 (0.006)**		12

**B**					
***Baseline perfusion***					
sBAT	+ 0.55 (0.071)		+ 0.42 (0.178)		12
***Cold 1 perfusion***					
sBAT	+ 0.38 (0.227)		+ 0.37 (0.243)		12
***Reheated perfusion***					
sBAT	** + 0.78 (0.005)**		** + 0.65 (0.022)**		12

Correlation of measurements estimated within the same segmentation. ρ, Spearman rank-order correlation coefficient. r, Pearson correlation coefficient. n, number of observations. Statistical significance (*P* < 0.05) in bold font.

#### Changes in FF, perfusion and V_A_

**Figure 4:**
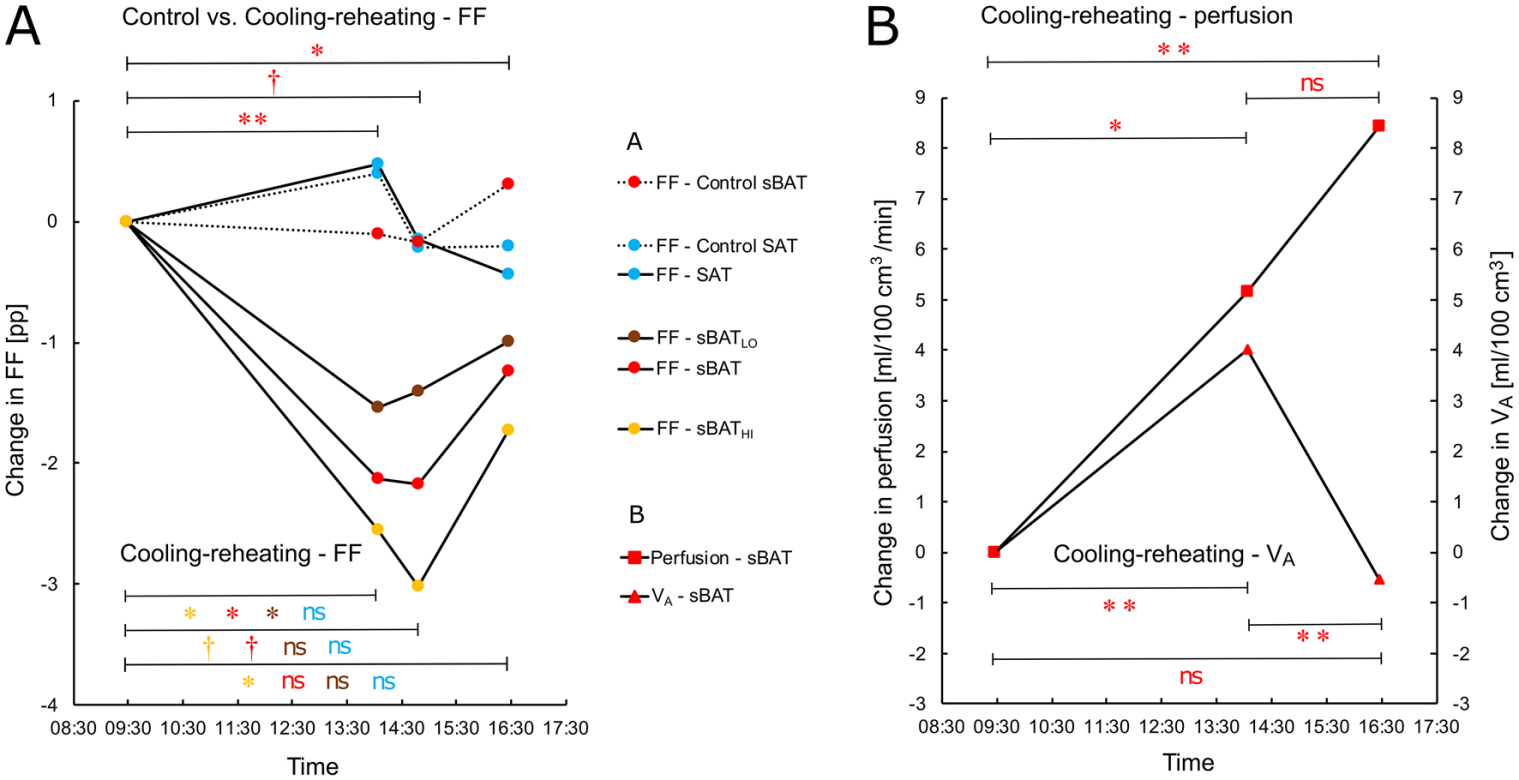
Changes in FF, perfusion and V_A_, within sBAT and SAT, with respect to baseline. **(A)** Changes in FF during the Cooling-reheating and Control protocols, with significance bars indicating changes during the Cooling-reheating protocol (at the bottom) and differences between the two protocols (at the top). **(B)** Changes in perfusion and V_A_ during the Cooling-reheating protocol indicated with significance bars. Measurements represent group mean values based on complete data available for estimating the changes between individual time points, i.e. different group mean values can be based on a different number of subjects. Solid line, Cooling-reheating protocol. Dotted line, Control protocol. Time, approximate mean session time for all subjects. pp, percentage points. Changes evaluated using Wilcoxon signed-rank test. Change between time points at **P < 0.01, *P < 0.05. Trend of change between time points at ^†^0.05 ≤ *P* < 0.10. ns, non-significant.

**Figure 5:**
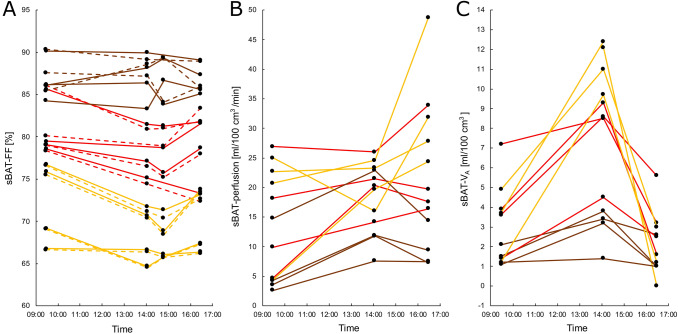
Changes in **(A)** FF, **(B)** perfusion and **(C)** V_A_ within sBAT of individual subjects over time during the Cooling-reheating protocol. Solid lines, sBAT. Dashed lines, sBAT_HI_. Yellow, red and brown correspond to the four subjects with the highest, intermediate and lowest sBAT-MR_glu_, respectively. In the sBAT-FF measurement, cold scan 1 was missing for one subject and cold scan 2 for two other subjects. A fourth subject did not show any sBAT_HI_. In the sBAT-V_A_ measurement, the baseline scan was missing for one subject and the reheated scan for another subject. Time, approximate mean session time for all subjects.

**Figure 6:**
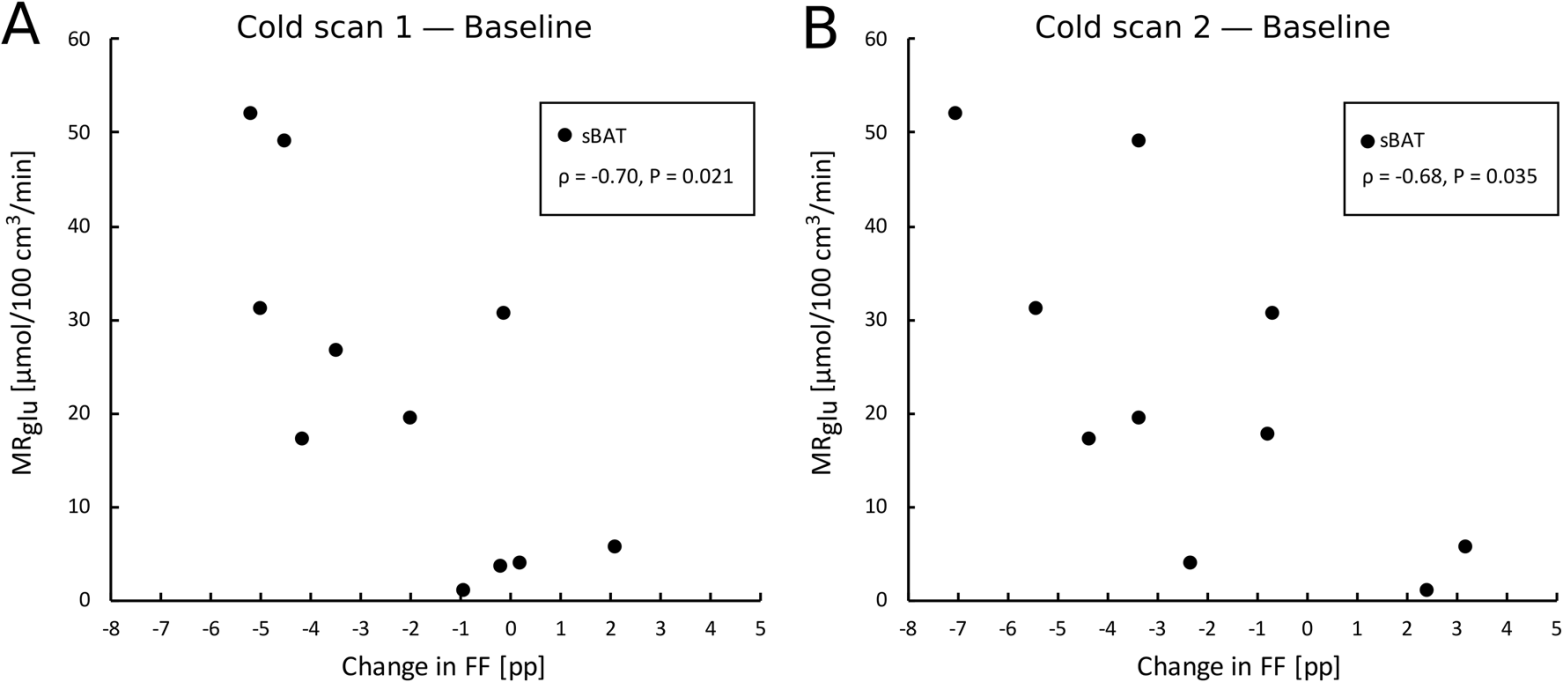
Correlation between cold-induced MR_glu_ and changes in FF. sBAT-MR_glu_ vs. the change in sBAT-FF between baseline and **(A)** ~ 3 h of cold exposure (cold scan 1) and **(B)** ~ 4.5 h of cold exposure (cold scan 2). ρ, Spearman rank-order correlation coefficient.

Changes in FF, perfusion and V_A_ in sBAT, sBAT_HI_, sBAT_LO_ and SAT are presented in Table 3A,B,C and Figs. 4, 5 and 6. During cold exposure (cold scan 1), FF in all regions of sBAT decreased by ~ 2 pp compared to baseline. The decreases in sBAT_HI_ and sBAT_LO_ were not significantly different from each other (*P* = 0.105). In SAT, there was no observable change in FF. sBAT-perfusion increased by 5.2 ml/100 cm^3^/min and V_A_ by 4.0 ml/100 cm^3^ during cold exposure.

**Table 3:** Changes in **(A)** FF within sBAT and SAT, **(B)** perfusion and **(C)** V_A_ within sBAT, presented side by side with the correlation between these changes and MR_glu_ and ΔFF (estimated within the same segmentation, correlation with ΔFF only conducted for perfusion and V_A_), during the Cooling-reheating protocol. **(A)** also presents changes in FF during the Control protocol and whether these changes are different from those of the Cooling-reheating protocol.

ΔFF variable	Cooling-reheating protocol					Control protocol		Cooling-reheating vs. Control		
	ΔFF *(P)*		ρ(MR_glu_) *(P)*		n	ΔFF	n	ΔFF diff *(P)*		n
**A**										
***Cold1–Baseline***										
sBAT	**− 2.13 ± 2.49 (0.024)**		**− 0.70 (0.021)**		11	**− **0.11 ± 1.43	*10*	**− 2.45 ± 2.51 (0.010)**		10
sBAT_HI_	**− 2.55 ± 2.84 (0.020)**		**− **0.64 (0.054)		10	···		···		
sBAT_LO_	**− 1.54 ± 2.33 (0.042)**		**− **0.36 (0.273)		11	···		···		
SAT	+ 0.47 ± 0.87 (0.148)		···		11	+ 0.39 ± 1.33	10	···		
***Cold2–Baseline***										
sBAT	**− **2.17 ± 3.26 (0.064)		**− 0.68 (0.035)**		10	**− **0.17 ± 1.12	10	**− **2.92 ± 3.25 (0.078)		8
sBAT_HI_	**− **3.02 ± 3.32 (0.055)		**− **0.52 (0.162)		9	···		···		
sBAT_LO_	**− **1.40 ± 2.83 (0.193)		**− **0.39 (0.263)		10	···		···		
SAT	**− **0.15 ± 0.97 (0.770)		···		10	**− **0.22 ± 1.03	10	···		
***Cold2–Cold1***										
sBAT	**− **0.13 ± 1.78 (0.652)		···		9	**− **0.03 ± 1.33	11	···		
sBAT_HI_	**− **0.70 ± 1.41 (0.313)		···		8	···		···		
sBAT_LO_	+ 0.12 ± 1.66 (0.910)		···		9	···		···		
SAT	**− 0.69 ± 0.37 (0.004)**		···		9	**− **0.56 ± 0.80	11	···		
***Reheat–Baseline***										
sBAT	**− **1.24 ± 2.24 (0.129)		**− **0.45 (0.147)		12	+ 0.30 ± 1.53	10	**− 2.13 ± 2.08 (0.020)**		10
sBAT_HI_	**− 1.72 ± 2.47 (0.032)**		···		11	···		···		
sBAT_LO_	**− **0.99 ± 1.71 (0.176)		···		12	···		···		
SAT	**− **0.43 ± 0.92 (0.204)		···		12	**− **0.20 ± 0.89	10	···		
***Reheat–Cold1***										
sBAT	+ 0.58 ± 1.72 (0.320)		+ 0.45 (0.163)		11	+ 0.67 ± 1.46	11	··· ···		
sBAT_HI_	+ 0.32 ± 1.99 (0.695)		···		10	···		···		
sBAT_LO_	+ 0.54 ± 1.73 (0.365)		···		11	···		···		
SAT	**− 0.80 ± 0.75 (0.014)**		···		11	**− **0.64 ± 1.04	11	···		
***Reheat–Cold2***										
sBAT	+ 1.32 ± 2.00 (0.084)		** + 0.65 (0.049)**		10	+ 0.70 ± 1.31	11	···		
sBAT_HI_	+ 1.74 ± 2.53 (0.074)		···		9	···		···		
sBAT_LO_	+ 0.55 ± 1.86 (0.432)		···		10	···		···		
SAT	**− **0.29 ± 1.06 (0.432)		···		10	**− **0.08 ± 1.03	11	···		

ΔPerfusion variable	Cooling-reheating protocol									
	ΔPerfusion *(P)*		ρ(MR_glu_) *(P)*		n	ρ(ΔFF) *(P)*		n		
**B**										
***Cold–Baseline***										
sBAT	** + 5.2 ± 6.7 (0.034)**		**− **0.42 (0.177)		12	+ 0.15 / + 0.03 (0.654 / 0.946)		11 / 10		
***Reheat–Baseline***										
sBAT	** + 8.4 ± 8.1 (< 0.001)**		** + 0.62 (0.037)**		12	− 0.14 (0.667)		12		
***Reheat–Cold***										
sBAT	+ 3.3 ± 9.2 (0.424)		** + 0.77 (0.005)**		12	+ 0.31 / + 0.50 (0.356 / 0.143)		11/ 10		

ΔV_A_ variable	ΔV_A_ *(P)*		ρ(MR_glu_) *(P)*		n	ρ(ΔFF) *(P)*		n		
**C**										
***Cold–Baseline***										
sBAT	** + 4.0 ± 2.9 (< 0.001)**		** + 0.83 (0.003)**		11	**− 0.81 (0.008 / 0.011)**/ **− 0.82**		10 / 9		
***Reheat–Baseline***										
sBAT	**− **0.5 ± 1.8 (0.407)		**− **0.01 (1.000)		10	+ 0.14 (0.707)		10		
***Reheat–Cold***										
sBAT	**− 4.8 ± 3.8 (< 0.001)**		**− 0.85 (0.002)**		11	**− **0.45 / **− **0.32 (0.191 / 0.410)		10 / 9		

Mean ± standard deviation. Δ, change between two time points during either protocol, calculated by subtracting the earlier measurement from the later: diff, difference calculated by subtracting the Control protocol measurement from the Cooling-reheating protocol measurement: /, separation between changes in FF calculated with respect to cold scan 1 (left) and cold scan 2 (right). Units: ΔFF, percentage points (pp); Δperfusion, ml/100 cm^3^/min; ΔV_A_, ml/100 cm^3^. ···, not estimated. n, number of observations. Changes were evaluated using Wilcoxon signed-rank test. Spearman rank-order correlation was used between MR_glu_ (or ΔFF) and changes in FF, perfusion and V_A_. ρ, Spearman rank-order correlation coefficient. Statistical significance (*P* < 0.05) in bold font.

During prolonged cold exposure (cold scan 2), there was a trend of decreased sBAT-FF relative to baseline (− 2.17 pp, *P* = 0.064). In sBAT_HI_, the trend was strong (− 3.02 pp, *P* = 0.055) and significantly different from that in sBAT_LO_, which was non-significant (*P* = 0.039 between changes in sBAT_HI_ and sBAT_LO_). SAT-FF did not change between baseline and cold scan 2. Between cold scan 1 and 2, the only significant change observed in FF was a decrease in SAT-FF (− 0.69 pp).

During reheating, sBAT-FF tended to normalize compared to during cold exposure (*P* = 0.129 vs. baseline; *P* = 0.320 vs. cold scan 1; *P* = 0.084 vs. cold scan 2) (Fig. 4A). sBAT_HI_-FF remained low (− 1.72 pp, *P* = 0.032 vs. baseline; + 0.32 pp, *P* = 0.695 vs. cold scan 1) despite a trend of normalization between prolonged cold exposure and reheating (+ 1.74 pp, *P* = 0.074 vs. cold scan 2). sBAT_LO_-FF did not change significantly relative to previous time points. The only change in SAT-FF between reheating and previous time points was a 0.80 pp decrease with respect to cold scan 1. sBAT-perfusion remained elevated during reheating (+ 8.4 ml/100 cm^3^/min, *P* < 0.001 vs. baseline; + 3.3 ml/100 cm^3^/min, *P* = 0.424 vs. cold exposure). sBAT-V_A_ normalized during reheating (− 0.5 ml/100 cm^3^, *P* = 0.407 vs. baseline; − 4.8 ml/100 cm^3^, *P* < 0.001 vs. cold exposure).

Correlations between MR_glu_ and changes in FF within sBAT, during the Cooling-reheating protocol, are presented in Table 3A and Fig. 6. Changes in sBAT-FF between baseline and cold scan 1 and between baseline and cold scan 2 correlated negatively with MR_glu_ (ρ ≤ − 0.68). In sBAT_HI_, however, there were only trends of correlations or non-significant correlations with MR_glu_. Changes in sBAT-perfusion between baseline and reheating, and between cold exposure and reheating, correlated positively with sBAT-MR_glu_ Table 3B. Changes in sBAT-V_A_ between baseline and cold exposure correlated positively with sBAT-MR_glu_ and changes in sBAT-V_A_ between cold exposure and reheating correlated negatively with sBAT-MR_glu_ Table 3C. In addition, changes in sBAT-V_A_ and sBAT-FF correlated negatively with each other, but only between baseline and cold exposure Table 3C.

### Control protocol

Baseline sBAT-FF and SAT-FF did not differ between the Cooling-reheating protocol and the Control protocol (Table S3, Supplemental material). The coefficient of variation between the baseline FF measurements of the two protocols was sBAT-CV = 1% and SAT-CV = 0.9%. Comparisons between the two protocols, regarding changes in sBAT-FF, are shown in Table 3A and Fig. 4A. The changes in sBAT-FF were larger or tended to be larger during the Cooling-reheating protocol compared to the Control protocol.

## Discussion

Safe and reliable imaging methods for quantification of BAT with respect to metabolic activity, lipid content and perfusion are highly warranted within the BAT research community for improving the feasibility of meticulous longitudinal studies in e.g. healthy adults and children. This study showed water-fat MRI to be a potential technique for assessment of cold-induced MR_glu_ of BAT by strong negative correlations between this estimate and FF of the anatomically defined sBAT depot, regardless of the temperature condition for the FF estimation, and low coefficients of variation between repeated FF measurements. These results indicate sBAT-FF, estimated either during warm or cold conditions, to be able to predict cold-induced MR_glu_ in BAT, which support previous studies^23,24,26,27^ and show higher correlation coefficients than the prior studies. Two possible reasons for the higher correlation coefficients obtained in the present study might be that the measurements during warm and cold conditions were performed on the same day instead of separate days^23,24^ and the use of the physiologically quantitative MR_glu_ as opposed to the semi-quantitative SUV^26,27^ to represent BAT glucose metabolism. The correlation coefficients were also stronger than those previously published by our research group from the same cohort, but where total sBAT-MR_glu_ (i.e. summed over all sBAT voxels) was used in linear regression analysis together with baseline sBAT-FF^30^, suggesting mean FF to be more suitable for prediction of mean than total MR_glu_ of BAT. However, our results diverge from those of three other studies, reporting no correlation during different temperature conditions^14,21,22^. The discordance is possibly related to the choice of representing BAT glucose metabolism by semi-quantitative estimates, instead of quantitative, and/or to the choice of studying BAT glucose metabolism during warm conditions. The difference between the studies might also be related to MR protocol design and signal model. In addition to associations within sBAT, this study demonstrated negative correlations between SAT-FF and SAT-MR_glu_. Although weaker compared to those of sBAT, they sustained for all temperature conditions of the FF measurement and might be due to e.g. considerable amounts of brown adipocytes being present in SAT and/or to non-BAT related properties. Such properties could be e.g. a relatively strong positive correlation between sBAT-FF and SAT-FF (Fig. S1B, Supplemental material) combined with a negative association between sBAT-FF and glucose tolerance, as previously observed in adolescents by our research group^34^. The relationship between SAT-FF and SAT-MR_glu_ might also be influenced by technical limitations such as differences in spatial resolution and partial volume effects between PET and MRI or to subjects moving during image acquisition, all leading to slight mismatches between the MR_glu_ and FF measurements.

Within sBAT at baseline, there was no difference between sBAT_HI_-FF and sBAT_LO_-FF and both subregions showed negative correlations with MR_glu_. During cold exposure (cold scan 1), sBAT_HI_-FF was lower compared to sBAT_LO_-FF, which agrees with a recent study^27^ but disagrees with another, reporting no general difference between parts of the depot exhibiting high and low [^18^F]FDG-SUV^25^. During prolonged cooling (cold scan 2), there was a trend of lower sBAT_HI_-FF compared to sBAT_LO_-FF, which ceased during reheating. Overall, the results indicate regional differences in sBAT-FF related to MR_glu_, possibly due to a heterogenous brown adipocyte content. These regional differences were subtle, maybe because cold-induced increases in perfusion, blood volume and lipid consumption can occur within the whole depot despite higher MR_glu_ in some subregions than others. It could also have technical origins, e.g. differences in spatial resolution and partial volume effects between PET and MRI or to subjects moving during image acquisition, leading to mismatches between the MR_glu_ and FF measurements. The inconsistent results between our study and the previous PET/MRI study^25^ might be technology-related, e.g. due to the use of MR_glu_ vs. [^18^F]FDG-SUV and/or to differences in MRI protocol parameters.

At the end of ~ 3 h of cold exposure, a decrease in sBAT-FF was observed. This agrees with a previous 1.5 T MRI-only study by our research group, based on a similar cohort and Cooling-reheating protocol^11^, but more modest than other past work (− 2.9 pp/h^12^, − 4.7 pp/h^13^). The discrepancies could be related to differences in image acquisition, segmentation and/or cooling protocol. The decrease in sBAT_HI_-FF did not differ from that in sBAT_LO_-FF. sBAT-perfusion increased by ~ 40% and sBAT-V_A_ by ~ 150% during cold exposure, from initial low values. Increases in perfusion of up to ~ 110%^15–17^ and in V_A_ of ~ 70–210%^17^ have previously been observed by other groups. These increases reflect elevated blood supply by an increase in arterial blood volume possibly accompanied by an increase in blood flow velocity. During prolonged cold exposure (cold scan 2 in the present study), there was only a trend of decreased sBAT-FF compared to baseline, which was strong in sBAT_HI_ but negligible in sBAT_LO_. The change in sBAT_HI_ was larger than in sBAT_LO_. Some trends and non-significant results obtained during prolonged cold exposure seemingly contradicted those during initial cold exposure. However, inconsistencies could have resulted from the limited statistical power obtained from studying only 12 individuals. Overall, the results at least indicate a larger cold-induced decrease in sBAT_HI_-FF compared to sBAT_LO_-FF. During reheating, the cold-induced decrease in sBAT-FF tended to normalize. This observation differs from that of our previous 1.5 T MRI-only study, where a sustained low sBAT-FF was obtained during reheating^11^. The reasons for the discrepancy are not known. One hypothesis is that the longer reheating time in the present study (~ 1 h 20 min) was sufficient for enabling a noticeable replenishment of intracellular lipids in BAT and, as a consequence, an underestimation of lipid consumption compared to the previous study (reheating time ~ 30 min). To date, the knowledge of the time frame for intracellular lipid replenishment in human brown adipocytes during warm conditions, after cold expose, is very limited. Dedicated studies using fatty acid tracers undergoing esterification in the brown adipocytes, e.g. [^11^C]palmitate, could elucidate this research question. An alternative hypothesis is that the shorter reheating time of the previous study was too brief for normalization of the cold-induced increase in water content, related to increased arterial blood volume, as opposed to the present study.

The Cooling-reheating protocol is based on the following assumptions^11^: 1) BAT perfusion (and associated blood volume) is relatively rapidly regulated and therefore likely to regress by short reheating after cold exposure. 2) Lipid content is relatively slowly regulated and therefore not regressing during the same reheating time. The first assumption of fast normalization of perfusion was not supported by the present study as sBAT-perfusion remained elevated during reheating. However, sBAT-V_A_, a parameter more directly related to sBAT water content and sBAT-FF than sBAT-perfusion, normalized during the same conditions (note the longer reheating time in the present vs. the original study^11^). In the present study, there was no significant decrease in sBAT-FF between baseline and reheating. However, this result was based on subjects with a relatively large spread of mean sBAT-MR_glu_. When considering sBAT_HI_ only, the decrease between baseline and reheating was significant. Altogether, these results indicated the decrease in sBAT_HI_-FF, observed during reheating (vs. baseline), to likely be due to lipid consumption. However, the influence of perfusion and blood volume, on the changes in FF observed during cold exposure, could not be ruled out. These conclusions were supported by a significant negative correlation between the changes in sBAT-V_A_ and sBAT-FF, measured between baseline and cold exposure, but not between the changes measured between baseline and reheating or between cold exposure and reheating. To our knowledge, this study is the first to apply repeated PET perfusion measurements during a Cooling-reheating protocol for determining the changes in FF as being related to perfusion (and blood volume) or lipid consumption. The reason for the contradictory pattern of sustained high sBAT-perfusion and normalized sBAT-V_A_ during reheating is not known. The observation could reflect a normalization of the number of blood-supplying arterioles and capillaries in sBAT concomitant with an increase in blood flow velocity in remaining vessels, representing a residual effect of the cold-induced increase in metabolic turnover.

Between the time points of baseline and each of the succeeding three scans, the changes in sBAT-FF were significantly larger, or tended to be larger, during the Cooling-reheating protocol compared to the Control protocol. This supports the notion that the changes, observed during the temperature intervention, were associated with cold-induced BAT MR_glu_ and not with other study-related procedures, e.g. the length of fasting.

In both warm and cold conditions, sBAT-FF was lower compared to SAT-FF, which agrees with the literature^20,21,23,35^. SAT-FF did not change between baseline and cold exposure but decreased between cold scan 1 and 2, likely due to the supine position of the subjects in-between the two scans as described previously^11^. It can be concluded that SAT-FF exhibits a different pattern than sBAT-FF during cooling and reheating, which is visible in Fig. 4A. The difference is probably related to the tissue contents of brown adipocytes but might also be non-BAT-related, e.g. due to the study procedure involving a supine position of the subjects during scanning. The present study showed slightly higher estimates of perfusion but similar estimates of V_A_ as previous studies^15–17^ but a lack of association between perfusion and MR_glu_ during cold exposure, as opposed to one of these previous studies^15^. The reason for this disparity is not known. sBAT-perfusion and sBAT-V_A_ during reheating were estimated approximately one half-life of ^18^F after the administration of [^18^F]FDG. However, residual [^18^F]FDG is not expected to have influenced the perfusion and V_A_ results considerably as this was corrected for.

This study has some shortcomings. The small cohort led to limited statistical power, possibly causing type II errors. Such errors could be responsible for some seemingly ambiguous results, e.g. sBAT-FF decreased between baseline and cold scan 1 (− 2.13 pp, *P* = 0.024, *n* = *11*) but only tended to decrease between baseline and cold scan 2 (− 2.17 pp, *P* = 0.064, *n* = *10*). Another related limitation was that of missing data, which resulted in statistical analyses based on different subjects at different time points. The cooling protocol aimed at similar cooling conditions in all individuals, at a level slightly above the self-reported limit of shivering. However, as shivering was only subjectively assessed and as shivering in e.g. deep muscles can be difficult or impossible to perceive, an inter-subject variability in the cold stimulus directly affecting MR_glu_ was likely to have occurred, which is a limitation that needs to be emphasized. An individualized cooling protocol, standardized with respect to objective measurements of heat loss (by e.g. skin temperature) and heat production (by e.g. indirect calorimetry), could more reliably have ensured an adequate level of cold stimulation of BAT in each individual and more equal and repeatable cooling conditions of the subjects. Moreover, reheating was performed according to a fixed procedure, without attempts of individual adaption and without objective measurements, also leading to inter-subject differences in the amount of reheating experienced. Although more objective and personalized settings would have been preferred, they were considered as difficult to fit into the already comprehensive study protocol and were therefore omitted. The glucose analogue tracer [^18^F]FDG is well-established for in vivo imaging of human BAT, with previous studies indicating positive relationships between cold-induced glucose metabolism and NST in BAT^24,29^. Such relationships are exemplified by e.g. a positive correlation between cold-induced BAT [^18^F]FDG-SUV_max_ and the cold-induced increase in energy expenditure^29^, a lower cold-induced decrease in supraclavicular skin temperature of subjects with visually detectable compared to non-detectable BAT (from [^18^F]FDG-PET)^29^, and a positive correlation between the difference in cold-induced MR_glu_ between BAT and WAT and the cold-induced temperature increase in BAT (assessed with MRS)^24^. Despite these relationships, [^18^F]FDG does not explicitly measure the thermogenic state of BAT (i.e. NST), for which oxidative metabolism tracers such as [^15^O]O_2_ and [^11^C]acetate could be suitable alternatives. However, logistic and technical challenges associated with these tracers make them difficult to use in already complex and lengthy protocols such as in this study. No correction for multiple comparison was applied to the results, which might have led to type I errors. However, when multiple tests were performed on similar variables and their results pointed to the same conclusions and/or were in line with the literature, the results were considered as complementary rather than as probable random findings.

A strength of this study was the possibility of simultaneous MRI and PET within a single session. Despite this opportunity, the entire study protocol was long and challenging for the subjects, which likely influenced study compliance. Slight misalignments between MRI and PET within one session were observed and reduced by rigid image registration (see “Image post-processing” section). Another strength of this study was the inclusion of a control protocol, which enabled the changes in FF to be determined as being induced by temperature and not by other study-related procedures. As measurements during warm and cold conditions were performed within the same day in the present study, potential influence from day-to-day or long-term alterations in BAT could be avoided.

## Conclusion

Mean FF of the anatomically defined sBAT depot could be used to predict its cold-induced BAT metabolic rate of glucose, regardless of temperature condition preceding the FF estimation. The Cooling-reheating protocol was shown useful for studying changes in the lipid concentration biomarker sBAT-FF, related to warm and cold conditions. The FF decreases observed at the end of reheating were mainly due to lipid consumption, but could potentially be underestimated due to intracellular lipid replenishment. The influence of perfusion and blood volume, on the changes in FF observed during cold exposure, could not be ruled out.

## Supplementary Information

Supplementary Information.

## Data Availability

The data sets generated and analysed during the current study are available from the corresponding author at reasonable request, unless the requested sharing is conflicting with the approved ethics application.
