# Supplementary material for: PET/MRI of glucose metabolic rate, lipid content and perfusion in human brown adipose tissue
Source: Sci Rep. 2021 Jul 22;11:14955. doi: 10.1038/s41598-021-87768-w (PMC8298487; doi:10.1038/s41598-021-87768-w)
Supplement: Supplementary file 1 — Supplementary Information. [file 41598_2021_87768_MOESM1_ESM.pdf]

Supplemental material

PET/MRI of glucose metabolic rate, lipid content and perfusion in human brown adipose tissue

Elin Lundström[1], Jonathan Andersson[1], Mathias Engström[2], Mark Lubberink[1,3], Robin Strand[1,4], Håkan Ahlström[1,5], Joel Kullberg[1,5]

[1] Department of Surgical Sciences, Section of Radiology, Uppsala University, Uppsala, Sweden
[2] GE Healthcare, Waukesha, Wisconsin, USA
[3] Medical Physics, Uppsala University Hospital, Uppsala, Sweden
[4] Department of Information Technology, Uppsala University, Uppsala, Sweden
[5] Antaros Medical, BioVenture Hub, Mölndal, Sweden

Correspondence
E-mail: elin.lundstrom@radiol.uu.se

....

Materials and methods

Mean fat fraction (FF) of suspected BAT (sBAT-FF) was estimated from segmentations obtained with a modified version of a previously presented method[1], implemented in MATLAB. The method was atlas-based with the atlas sBAT depots defined according to anatomical criteria. The main adjustments to the published method are provided below in *1) Generation of atlases – 4) Fine adjustment and refinement of segmentations*.

1) Generation of atlases – Initially, an atlas was created from the baseline scan of each subject (denoted *atlas 1*). The atlas consisted of a water signal image and a fat signal image (instead of a water fraction and an R_2* map as previously described[1]) with an associated crude sBAT volume of interest (VOI) segmentation. Prior to image registration, pre-processing of the water and fat signal images was carried out, which aimed for removal of voxels outside the body (background and water piping). Segmentation of the crude sBAT VOI was performed manually from anatomically defined criteria as previously described[1]. The two outmost slices, both in superior and inferior direction of the image data, were removed to reduce possible effects related to non-optimal slab profiles at the edges of the 3D image volume.

2) Image registration – A single-atlas approach, instead of the previously described multi-atlas approach[1], was used. Prior to image registration, the water and fat signal images of the rest of the scans of each subject were pre-processed as described above for the baseline scan in *Generation*

of atlases. For each subject, a pairwise intra-subject image registration between atlas 1 and cold scan 1 (target) was carried out in line with the previously described method[1]. An initial affine registration was performed between the water signal images and a subsequent elastic registration was performed between both the water and fat signal images.

3) Propagation of segmentations – For each pairwise image registration, the resulting deformation field was applied to transfer the crude sBAT VOI from atlas 1 to the target. Two additional crude segmentations were created from the deformed crude sBAT VOI and the metabolic rate of glucose (MR_{glu}) map. A crude high MR_{glu} sBAT VOI (crude $sBAT_{HI}$) was obtained by including only sBAT VOI voxels with MR_{glu} > 11 µmol/100 cm^3/min. The crude low MR_{glu} sBAT VOI (crude $sBAT_{LO}$) contained sBAT VOI voxels with $MR_{glu} \leq 11$ µmol/100 cm^3/min. The crude sBAT, $sBAT_{HI}$ and $sBAT_{LO}$ segmentations together with the water and fat signal images of cold scan 1 formed a new single atlas (denoted *atlas 2*) that was used to automatically segment the rest of the scans from both visits, using the same registration method as described above in *Image registration*. The MR_{glu} threshold limit of 11 µmol/100 cm^3/min aimed to separate active sBAT and inactive subcutaneous adipose tissue (SAT), and was determined empirically.

4) Fine adjustment and refinement of segmentations – After the crude segmentations had been transferred to all targets, an automated fine adjustment was applied to the crude sBAT VOI, for isolating the adipose tissue and reducing the partial volume effects from adjacent non-adipose tissues. This fine adjustment comprised a voxel threshold on FF (≥ 40%), a six-neighbourhood erosion, a voxel threshold on R_2* (≤ 120 s^{-1}) and attempts to remove residual voxels in air and bone, based on thresholding on the sum of the water and fat signal images with subsequent steps of dilation, filling of holes and erosion. The threshold limits were determined empirically. In addition, the outmost slice, both in superior and inferior direction, was excluded from the segmentation. This fine adjustment was also applied to the crude $sBAT_{HI}$ and $sBAT_{LO}$ by merging the final sBAT VOI with the crude $sBAT_{HI}$ and $sBAT_{LO}$ VOIs. The final segmentations are referred to as sBAT, $sBAT_{HI}$ and $sBAT_{LO}$.

Posterior SAT was segmented from the same water-fat image slices used for segmenting sBAT, according to the following. A body mask was segmented from the background using thresholding on the sum of the water and fat signal images. The threshold level was manually adjusted if deemed necessary. Holes in the mask were filled. A muscle mask was created by removing voxels with R_2* > 100 s^{-1} (skin and bone) and FF ≥ 40% (adipose tissue and bone). A SAT mask was created by excluding voxels with FF < 40% from the body mask, and also by excluding voxels enclosed within the muscle mask to remove voxels in bones and non-SAT. The area superior of the centre of gravity of the body mask (in anterior direction), determined slice-wise, was removed from the SAT mask. A six-neighbourhood erosion was performed to decrease the partial volume effects. Voxels with R_2* > 120 s^{-1} (residual skin and bones) were removed from the SAT mask. Finally, some minor manual corrections were performed. Regions of high and low MR_{glu} within posterior SAT were obtained using the same threshold level (MR_{glu} = 11 µmol/100 cm^3/min) as for sBAT. The final segmentations are referred to as SAT, SAT_{HI} and SAT_{LO}.

Results

Comparison and correlation between mean sBAT-FF and SAT-FF, estimated at baseline for each subject, are shown in Fig. S1. Results from correlation of sBAT-FF and SAT-FF with MR_{glu} and MR_{glu2}, estimated within the same segmentation, are presented in Table S1. The MR_{glu} and MR_{glu2} were derived from different plasma glucose assessments, C_{glu}^{P} and C_{glu}^{P2}, respectively (see details in manuscript). The FF, perfusion, V_A, MR_{glu} and segmented volume of different subregions of sBAT and SAT, obtained from the Cooling-reheating and Control protocols, are summarized in Table S2.

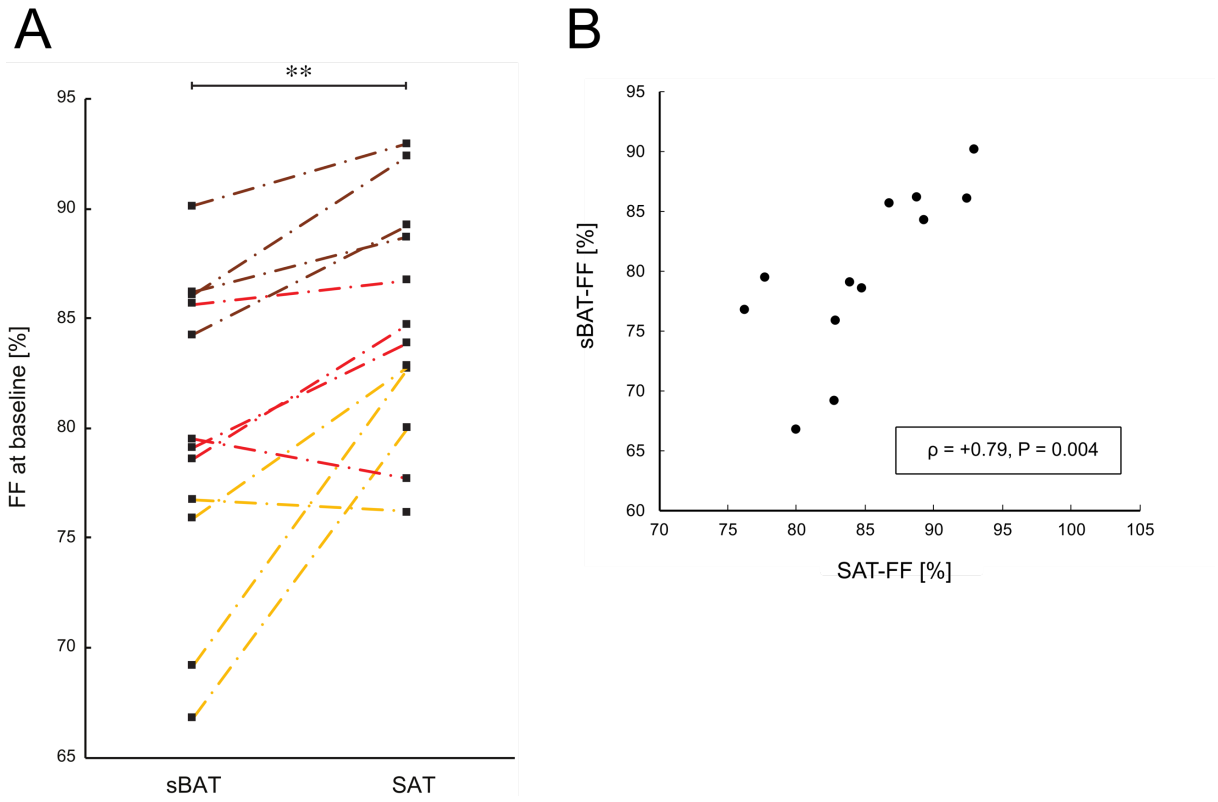

Figure S1. Baseline FF measurements from the Cooling-reheating protocol. **(A)** Mean sBAT-FF and SAT-FF for each individual. Yellow, red and brown indicate the four subjects with the highest, intermediate and lowest sBAT-MR_{glu}, respectively. Difference between sBAT and SAT at $^{**}P < 0.01$. **(B)** Correlation between sBAT-FF and SAT-FF. ρ, Spearman rank-order correlation coefficient.

	**Correlation with MR_{glu}**					**Correlation with MR_{glu2}**				
**Measurement**	**ρ**	***(P)***	**r**	***(P)***	***n***	**ρ**	***(P)***	**r**	***(P)***	***n***
**Baseline FF**										
sBAT	**-0.88**	***(< 0.001)***	**-0.81**	***(0.001)***	*12*	**-0.87**	***(< 0.001)***	**-0.85**	***(< 0.001)***	*11*
SAT	**-0.83**	***(0.002)***	**-0.71**	***(0.009)***	*12*	**-0.90**	***(< 0.001)***	**-0.71**	***(0.014)***	*11*
**Cold 1 FF**										
sBAT	**-0.89**	***(< 0.001)***	**-0.90**	***(< 0.001)***	*11*	**-0.91**	***(< 0.001)***	**-0.92**	***(< 0.001)***	*11*
SAT	**-0.80**	***(0.005)***	**-0.69**	***(0.020)***	*11*	**-0.87**	***(< 0.001)***	**-0.76**	***(0.006)***	*11*
**Cold 2 FF**										
sBAT	**-0.90**	***(< 0.001)***	**-0.91**	***(< 0.001)***	*10*	**-0.90**	***0.002***	**-0.93**	***(< 0.001)***	*9*
SAT	**-0.83**	***(0.006)***	**-0.69**	***(0.029)***	*10*	**-0.80**	***0.014***	**-0.70**	***(0.035)***	*9*
**Reheated FF**										
sBAT	**-0.87**	***(< 0.001)***	**-0.87**	***(< 0.001)***	*12*	**-0.85**	***0.002***	**-0.89**	***(< 0.001)***	*11*
SAT	**-0.83**	***(0.001)***	**-0.74**	***(0.006)***	*12*	**-0.88**	***(< 0.001)***	**-0.74**	***(0.009)***	*11*

Table S1. Correlation between FF and MR_{glu} and between FF and MR_{glu2} in sBAT and SAT during the Cooling-reheating protocol. ρ, Spearman rank-order correlation coefficient. r, Pearson correlation coefficient. *n*, number of observations. Statistical significance ($P < 0.05$) in bold font.

A

	Baseline			Cold 1			Cold 2			Reheated		
**Variable**	**sBAT**<br>***n=12***<br>***(n=10)***	**sBAT$_{HI}$**<br>***n=11***<br>***(n=9)***	**sBAT$_{LO}$**<br>***n=12***<br>***(n=10)***	**sBAT**<br>***n=11***<br>***(n=11)***	**sBAT$_{HI}$**<br>***n=10***<br>***(n=10)***	**sBAT$_{LO}$**<br>***n=11***<br>***(n=11)***	**sBAT**<br>***n=10***<br>***(n=11)***	**sBAT$_{HI}$**<br>***n=9***<br>***(n=10)***	**sBAT$_{LO}$**<br>***n=10***<br>***(n=11)***	**sBAT**<br>***n=12***<br>***(n=11)***	**sBAT$_{HI}$**<br>***n=11***<br>***(n=10)***	**sBAT$_{LO}$**<br>***n=12***<br>***(n=11)***
FF	79.8±7.1<br>(79.6±8.1)	79.5±7.5<br>(79.0±8.7)	80.8±5.9<br>(80.9±6.5)	77.7±8.7<br>(80.3±7.6)	76.9±9.2<br>(79.5±7.7)	79.6±7.2<br>(81.7±6.1)	76.8±8.5<br>(80.3±7.9)	75.4±8.5<br>(80.3±9.0)	78.6±6.8<br>(81.4±6.5)	78.6±7.7<br>(81.0±8.1)	77.8±7.9<br>(81.2±9.6)	79.9±6.3<br>(82.2±7.0)
Perfusion	13.2±9.3<br>*n=12*	*na*	*na*	18.3±5.9<br>*n=12*	*na*	*na*	*na*	*na*	*na*	21.6±12.4<br>*n=12*	*na*	*na*
V_A	2.9±2.0<br>*n=11*	*na*	*na*	7.3±3.8<br>*n=12*	*na*	*na*	*na*	*na*	*na*	2.1±1.5<br>*n=11*	*na*	*na*
MR_{glu}	*na*	*na*	*na*	21.5±17.1<br>*n=12*	28.7±14.4<br>*n=11*	5.9±2.2<br>*n=12*	*na*	*na*	*na*	*na*	*na*	*na*
Volume	42.5±31.4<br>(40.4±28.0)	17.0±12.0<br>(20.2±13.8)	26.9±37.2<br>(22.2±33.2)	45.5±36.7<br>(45.8± 32.3)	16.0±12.1<br>(18.5±13.7)	30.9±42.0<br>(29.0±38.7)	37.2±31.0<br>(45.5±33.2)	16.0±11.5<br>(17.9±12.3)	22.8±33.6<br>(29.2±39.7)	40.3±33.2<br>(45.9±33.7)	14.8±11.4<br>(18.4±13.2)	26.7±37.7<br>(29.2±40.1)

B

	Baseline			Cold 1			Cold 2			Reheated		
**Variable**	**SAT**<br>***n=12***<br>***(n=10)***	**SAT$_{HI}$**<br>***n=4***<br>***(n=3)***	**SAT$_{LO}$**<br>***n=12***<br>***(n=10)***	**SAT**<br>***n=11***<br>***(n=11)***	**SAT$_{HI}$**<br>***n=3***<br>***(n=3)***	**SAT$_{LO}$**<br>***n=11***<br>***(n=11)***	**SAT**<br>***n=10***<br>***(n=11)***	**SAT$_{HI}$**<br>***n=4***<br>***(n=3)***	**SAT$_{LO}$**<br>***n=10***<br>***(n=11)***	**SAT**<br>***n=12***<br>***(n=11)***	**SAT$_{HI}$**<br>***n=3***<br>***(n=3)***	**SAT$_{LO}$**<br>***n=12***<br>***(n=11)***
FF	84.8±5.4<br>(85.2±5.1)	79.5±3.7<br>(80.3±6.8)	84.9±5.4<br>(85.2±5.0)	86.0±5.0<br>(86.1±4.4)	79.5±1.9<br>(82.0±5.6)	86.0±4.9<br>(86.2±4.2)	83.9±5.1<br>(85.5±4.9)	80.4±3.4<br>(78.1±5.6)	84.0±5.0<br>(85.6±4.8)	84.4±5.8<br>(85.4±4.9)	78.2±2.6<br>(79.4±6.7)	84.5±5.7<br>(85.5±4.8)
MR_{glu}	*na*	*na*	*na*	3.6±1.6<br>*n=12*	13.6±3.6<br>*n=4*	3.4±1.2<br>*n=12*	*na*	*na*	*na*	*na*	*na*	*na*
Volume	130.1±114.7<br>(114.6±87.3)	2.8±5.6<br>(3.5±6.0)	129.2±115.2<br>(113.6±87.9)	140.4±119.8<br>(138.7±114.5)	4.0±6.8<br>(3.8±6.4)	139.3±120.4<br>(137.6±115.3)	116.7±100.8<br>(142.0±123.8)	2.9±5.7<br>(3.8±6.5)	115.5±101.3<br>(140.9±124.6)	123.7±110.2<br>(138.5±121.8)	3.9±6.7<br>(3.7±6.3)	122.7±110.8<br>(137.5±122.4)

Table S2. Summary of measurements in **(A)** sBAT and **(B)** SAT from the study population during the Cooling-reheating protocol, with corresponding measurements from the Control protocol in parenthesis when applicable. mean ± standard deviation. *n*, number of subjects or number of measurements in case measurements were lacking for some subjects. *na*, not applicable (measurements not conducted). Units: FF, %; perfusion, ml/100 cm^3/min; V_A, ml/100 cm^3, MR_{glu}, µmol/100 cm^3/min; volume, ml. Measurements were based on all subjects for which data were available and the segmented volume non-zero.

………

Comparisons between the Cooling-reheating and Control protocols are provided in Table S3. sBAT-FF and SAT-FF estimated at baseline did not differ between the protocols.

Variable	Cooling-reheating protocol	Control protocol	*P*	*n*
sBAT FF (*Baseline*)	79.25 ± 7.51	79.60 ± 8.09	*0.625*	*10*
SAT FF (*Baseline*)	84.79 ± 4.85	85.16 ± 5.07	*0.625*	*10*

Table S3. Differences in FF between the Cooling-reheating and Control protocols. mean ± standard deviation. *n*, number of observations used in statistical analyses. P values from Wilcoxon signed-rank test. Statistical significance ($P < 0.05$) in bold font.

……………………………………………………………………………………………………

Discussion

According to Table S1, FF and glucose metabolic rate (estimated by MR_{glu} and MR_{glu2}), within the whole sBAT and SAT depots, showed relatively strong negative correlations. The Spearman and Pearson correlation coefficients were similar for MR_{glu} and MR_{glu2}, although slightly stronger for MR_{glu2} overall. This could be due to the C_{glu}^{P2} measurement being more reliable and therefore more suitable than C_{glu}^{P} for estimating the metabolic rate of glucose. However, there was one missing data point for C_{glu}^{P2}, which further limits the statistical power in this cohort of 12 subjects. For this reason, C_{glu}^{P} and not C_{glu}^{P2}, was used in most analyses of the manuscript.

References

1 Lundström, E. *et al.* Automated segmentation of human cervical-supraclavicular adipose tissue in magnetic resonance images. *Sci Rep* **7**, 3064, doi:10.1038/s41598-017-01586-7 (2017).
